# Hydroquinone Induces NLRP3-Independent IL-18 Release from ARPE-19 Cells

**DOI:** 10.3390/cells10061405

**Published:** 2021-06-06

**Authors:** Niina Bhattarai, Eveliina Korhonen, Yashavanthi Mysore, Kai Kaarniranta, Anu Kauppinen

**Affiliations:** 1 School of Pharmacy, Faculty of Health Sciences, University of Eastern Finland, 70210 Kuopio, Finland; eveliina.korhonen@uef.fi (E.K.); yashavanthi.mysore@uef.fi (Y.M.); 2 Department of Clinical Chemistry, University of Helsinki and Helsinki University Hospital, 00290 Helsinki, Finland; 3 Department of Ophthalmology, Institute of Clinical Medicine, University of Eastern Finland, 70210 Kuopio, Finland; kai.kaarniranta@uef.fi; 4 Department of Ophthalmology, Kuopio University Hospital, 70210 Kuopio, Finland

**Keywords:** hydroquinone, oxidative stress, IL-1β, IL-18, NLRP3, RPE cell, PARP, DNA damage, NAC, APDC

## Abstract

Age-related macular degeneration (AMD) is a retinal disease leading to impaired vision. Cigarette smoke increases the risk for developing AMD by causing increased reactive oxygen species (ROS) production and damage in the retinal pigment epithelium (RPE). We have previously shown that the cigarette tar component hydroquinone causes oxidative stress in human RPE cells. In the present study, we investigated the propensity of hydroquinone to induce the secretion of interleukin (IL)-1β and IL-18. The activation of these cytokines is usually regulated by the Nucleotide-binding domain, Leucine-rich repeat, and Pyrin domain 3 (NLRP3) inflammasome. ARPE-19 cells were exposed to hydroquinone, and cell viability was monitored using the lactate dehydrogenase (LDH) and 3-(4,5-dimethylthiazol-2-yl)-2,5-diphenyltetrazolium bromide salt (MTT) assays. Enzyme-linked immunosorbent assays (ELISAs) were used to measure the levels of proinflammatory cytokines IL-1β and IL-18 as well as NLRP3, caspase-1, and poly (ADP-ribose) polymerase (PARP). Hydroquinone did not change IL-1β release but significantly increased the secretion of IL-18. Cytoplasmic NLRP3 levels increased after the hydroquinone treatment of IL-1α-primed RPE cells, but IL-18 was equally released from primed and nonprimed cells. Hydroquinone reduced the intracellular levels of PARP, which were restored by treatment with the ROS scavenger N-acetyl-cysteine (NAC). NAC concurrently reduced the NLRP3 levels but had no effect on IL-18 release. In contrast, the NADPH oxidase inhibitor ammonium pyrrolidinedithiocarbamate (APDC) reduced the release of IL-18 but had no effect on the NLRP3 levels. Collectively, hydroquinone caused DNA damage seen as reduced intracellular PARP levels and induced NLRP3-independent IL-18 secretion in human RPE cells.

## 1. Introduction

Cigarette smoke is a risk factor of age-related macular degeneration (AMD) that induces apoptosis, DNA damage, endoplasmic reticulum (ER) stress, mitochondrial fragmentation, reactive oxygen species (ROS) production and vascular endothelial growth factor (VEGF) expression in retinal pigment epithelial (RPE) cells [1,2,3,4,5]. RPE cells maintain retinal homeostasis and support the functionality of photoreceptor cells, i.e., these cells are essential for good vision [6,7,8]. AMD can develop either into a dry (85–90%) or wet (10–15%) disease form [6,7]. The late forms of AMD manifest with a geographic atrophy in dry AMD or with an exudative form in wet AMD [7,9,10]. RPE degeneration and inflammation are involved in both disease forms [7,11,12]. RPE cell degeneration is a typical finding, especially in atrophic AMD, whereas choroidal neovascularization, hemorrhages, and swelling of the retina are hallmarks of wet AMD [7,9,10]. Oxidative stress and nucleotide-binding domain, leucine-rich repeat, and pyrin domain 3 (NLRP3) inflammasome activation are fundamental in the pathogenesis of AMD [5,7,10,12,13,14].

Hydroquinone, a component included in cigarette smoke, induces oxidative stress but prevents nuclear factor kappa B (NF-ᴋB) activity and the release of interleukin (IL)-6 and IL-8 cytokines from RPE cells [5,15,16,17]. Increased ROS production is a known activator of NLRP3 inflammasome, leading to the cleavage and release of IL-1β with or without concurrent maturation of IL-18 in human RPE cells [14,18]. The NLRP3 receptor is a pattern recognition receptor (PRR) that senses a wide variety of danger signals (pathogen or damage-associated molecular patterns i.e., PAMPs or DAMPs, respectively) [12,19,20]. Along with ROS production, its activation mechanisms include lysosomal destabilization and adenosine triphosphate (ATP) release-associated purinergic receptor X7/P2X purinoceptor 7 (P2X7) activation, resulting in K^+^-efflux and Ca^2+^-influx [13,14,18,21,22,23]. NLRP3 inflammasome activation requires consecutive priming and activation signals [13,19,24]. The priming signal, typically mediated through a Toll-like receptor (TLR) or a cytokine receptor, leads to the expression of the NLRP3 protein as well as the proforms of IL-1β and caspase-1 [13,25,26,27]. Epithelial cells tend to express pro-IL-18 continuously without priming [25,27]. An activation signal induces the assembly of NLRP3 inflammasome complex where pro-caspase-1 becomes attached to oligomerised NLRP3 via the apoptosis-associated speck-like protein containing a caspase-recruitment domain (ASC). The formation of this complex results in caspase-1 activation and subsequent cleavage of IL-1β and IL-18 into their mature and secreted forms [12,19,20,28]. IL-1β is involved in inflammatory diseases but it also possesses angiogenic properties [29,30,31]. IL-18 is a pleiotropic cytokine with distinct effects in different AMD models [32,33]. In mice, it has been observed to be protective against tuberculosis but harmful in combatting *Ehrlichia* infection [34,35].

In this study, we investigated the propensity of hydroquinone to activate NLRP3 inflammasome signaling in human RPE cells since we have previously shown that hydroquinone can induce ROS production in ARPE-19 cells, and oxidative stress is one of the best-known mechanisms of NLRP3 inflammasome activation [14,15,18]. Our results show that in addition to DNA damage, hydroquinone induced both NLRP3 inflammasome and IL-1α priming-independent secretion of IL-18 in human ARPE-19 cells.

## 2. Materials and Methods

### 2.1. Cell Culture and Treatments

Experiments were performed using ARPE-19 cells (ATCC) that were cultured in DMEM/F12 (1:1) medium (Life Technologies, Paisley, UK) with added 10% fetal bovine serum (GE Healthcare Life Sciences, South Logan, UT, USA), penicillin 100 U/mL, streptomycin 100 μg/mL (Pen Strep, Life Technologies, Grand Island, NY, USA), and L-glutamine 2 mM (Life Technologies, Paisley, UK). The experiments were conducted in the same medium without serum supplement, and L-glutamine was added separately in each independent experiment just prior to the IL-1α-priming. Hydroquinone was dissolved in the medium before each experiment.

In the experiments, cells were seeded onto 12-well plates (Costar, Corning Inc., Kennebunk, ME, USA) at the density of 200,000 cells per well and incubated for three days at +37 °C under 5% CO_2_. The cells were first washed once using serum-free medium and then primed using recombinant human IL-1α (4 ng/mL, R&D Systems, Minneapolis, MN, USA) for 24 h at +37 °C and 5% CO_2_. Thereafter, the cells were washed once again with medium and exposed to hydroquinone (HQ, 10 μM to 500 μM; Sigma-Aldrich, Saint Louis, MO, USA) for 18 h at +37 °C and 5% CO_2_. After the treatments, medium samples were collected and centrifuged at 381× *g* and +4 °C for 10 min (Biofuge Fresco Heraeus Instruments, Newport Pagnell, UK). Cells were either used directly for a viability assay or collected and lysed for other analyses. Optimal hydroquinone concentrations were probed by exposing cells to 10–500 µM hydroquinone, and cell viability and the secretion of mature IL-1β and IL-18 were determined. Two hydroquinone concentrations, 10 μM and 200 μM, were selected for the subsequent NLRP3 assays using only 200 μM for ATP and caspase-1 measurements. Caspase-1 inhibitor (casp.-1 inhib.; 20 μM in DMSO 0.22% *v/v*, Ac-YVAD-cmk, Sigma, St. Louis, MO, USA) was added 1 h before hydroquinone exposure when the role of caspase-1 in IL-18 release was investigated. Hydroquinone (200 μM) was also tested on unprimed cells. In experiments of NLRP3 silencing, ARPE-19 cells were seeded onto 12-well plates at the density of 150,000 cells per well and incubated for 24 h at +37 °C and 5% CO_2_. Cells were washed once with DMEM/F12 medium without serum and antibiotics, and 500 μL of the same medium was added into each well. Cells were transfected by adding lipofectamine (lipofectamine RNAiMAX/lipofectamine 2000, Invitrogen by Thermo Life Technologies Corp., Carlsbad, CA, USA) with NLRP3 siRNA (siNLRP3 10 pmol; NLRP3 Silencer select predesigned siRNA, id:s41556, Ambion, Austin, TX, USA) diluted in Opti-MEM medium (Thermo Fisher Scientific, Grand Island, NY, USA) for 24 h at +37 °C and 5% CO_2_. Negative siRNA (Silencer select Negative control #1 siRNA, Ambion, Austin, TX, USA) and transfection reagent (Tr. reag.; Opti-MEM with lipofectamine) without siRNAs served as controls. After transfection, cells were washed, primed with IL-1α, and exposed to 200 μM hydroquinone, as described above. The effects of siNLRP3 transfection on the release of IL-18 and lactate dehydrogenase (LDH) were determined.

When determining the effects of antioxidants, cells were primed with IL-1α for 24 h at +37 °C and 5% CO_2_, washed, and exposed to the NADPH oxidase inhibitor ammonium pyrrolidinedithiocarbamate (APDC, 2 μM; Sigma-Aldrich, St. Louis, MO, USA) for 5 min, or to the ROS scavenger N-acetyl-cysteine (NAC, 5 mM; Sigma-Aldrich, St. Louis, MO, USA) for 1 h as in our previous investigation of hydroquinone-mediated ROS production [15]. Thereafter, 200 μM hydroquinone was added for an additional 18 h. Untreated and IL-1α-primed cells served as controls.

### 2.2. Cell Viability Assays

Before sample collection, the cells were visually observed and photographed using an Axio Vert A1 Zeiss microscope with AxioCam MRm camera and Zen 2011 program (Carl Zeiss Microscopy GmbH, Jena, Germany). Thereafter, half (500 μL) of the cell culture medium was collected and centrifuged, as described above. LDH release was measured from medium samples immediately after the collection using the CytoTox96^®^ Non-Radioactive Cytotoxicity Assay (Promega, Madison, WI, USA) according to the manufacturer’s instructions. Absorbance values were measured at a wavelength of 490 nm with a spectrophotometer BioTek, ELx808 and the Gen-5 2.04 program (Instruments Inc., Winooski, VT, USA).

3-(4,5-dimethylthiazol-2-yl)-2,5-diphenyltetrazolium bromide salt (MTT; 0.5 mg/mL, Sigma-Aldrich, St. Louis, MO, USA) was added to cells remained on 12-well culture plates in the rest of medium (500 μL) and incubated for 3 h in the dark at +37 °C and 5% CO_2_, as described previously [15]. Absorbance values were measured at the wavelength of 560 nm using the spectrophotometer BioTek, ELx808.

### 2.3. Enzyme-Linked Immunosorbent Assay

The enzyme-linked immunosorbent assay (ELISA) technique was used to determine the levels of active IL-1β and IL-18, intra- and extracellular NLRP3, as well as cleaved caspase-1 (p20) and poly (ADP-ribose) polymerase (PARP) levels. IL-1β, IL-18, NLRP3, and caspase-1 were measured using the IL-1β BD OptEIA^TM^ Human ELISA Kit (BD Biosciences, San Diego, CA, USA), the Human IL-18 ELISA kit (Invitrogen by Thermo Fisher Scientific, Vienna, Austria), the Human NACHT, LRR and PYD domains-containing protein 3 (NLRP3/c1orf7/CIAS1/NALP3/PYPAF1) ELISA kit (Cusabio Biotech co., LTD, Wuhan, Hubei Province, China), and the Quantikine ELISA Human caspase-1/ICE immunoassay (R&D systems, McKinley, MN, USA), respectively. Absorbance values were determined at the wavelength of 450 nm with the correction wavelength of 655 nm in a spectrophotometer (Bio-Rad Model 550 with the Microplate Manager 5.2 program, Bio-Rad Laboratories Inc., Hercules, CA, USA). DNA damage was determined by measuring the PARP levels with the PARP/Apoptosis Colorimetric Assay Kit (R&D Systems, Minneapolis, MN, USA) and detecting absorbance values at the wavelength of 450 nm using a microplate reader Bio-Rad Model 550.

For the detection of intracellular NLRP3, cells were lysed with 40 μL of 1× lysis buffer (Cell Lysis Buffer 10×, Cell Signaling Technology, Leiden, Netherlands) in a tube containing cells from one well of a 12-well plate and incubated on ice for 5 min. Thereafter, the lysates were sonicated (3 × 10 s) and centrifuged at 16,090× *g* and +4 °C for 10 min. Supernatants were moved into clean microtubes and their protein concentrations were measured utilizing a protocol based on the Bradford method [36]. Briefly, the bovine serum albumin fraction V (BSA 1 mg/mL; Roche, Mannheim, Germany) was used to create a standard curve (0.5–3.5 μg/μL). Thereafter, samples were pipetted (0.5–3.5 μL) into wells of a 96-well plate, Bradford solution (200 μL) was added to all wells, and absorbance values were measured at the wavelength of 595 nm in a spectrophotometer Bio-Rad Model 550. NLRP3 levels were determined from remaining cell lysate using the ELISA technique with the results normalized to the protein level of the respective sample.

When measuring the levels of cleaved caspase-1, cells were lysed using the RIPA buffer (Thermo Scientific, Rockford, IL, USA), incubated for 5 min on ice, and centrifuged at 16,090× *g* and +4 °C for 10 min. The Pierce bicinchoninic acid (BCA) protein Assay Kit (Thermo Scientific, Rockford, IL, USA) was used to measure protein concentrations, and 15 or 25 μg of protein from each sample was used for the ELISA analysis.

When measuring the PARP levels, cells were lysed according to the manufacturer’s protocol, and the Pierce BCA protein assay was used to determine protein levels. Thereafter, 5 μg of protein from sample was used for the ELISA assay.

### 2.4. Extracellular ATP

The levels of ATP in culture medium were measured using the CellTiter-Glo Luminescent Cell Viability Assay (Promega, Madison, WI, USA) following the manufacturer’s instructions. Luminescence values were measured using the BioTek Cytation3 imaging reader with the Gen-5 3.03 program (Instruments Inc., Winooski, VT, USA).

### 2.5. Statistical Analyses

Results were analyzed using the GraphPad Prism program 7.04 (Graphpad Software, San Diego, CA, USA). Normality was determined using the Shapiro-Wilk and D’Agostino & Pearson normality tests (data not presented). Statistical differences between all groups were determined with the Kruskal-Wallis test, and post hoc comparisons using the Mann-Whitney U-test. Results are shown as mean ± standard error of mean (SEM) and differences were considered as statistically significant with *p*-values below 0.05.

## 3. Results

### 3.1. Hydroquinone Induces IL-18 Secretion in ARPE-19 Cells

**Figure 1:**
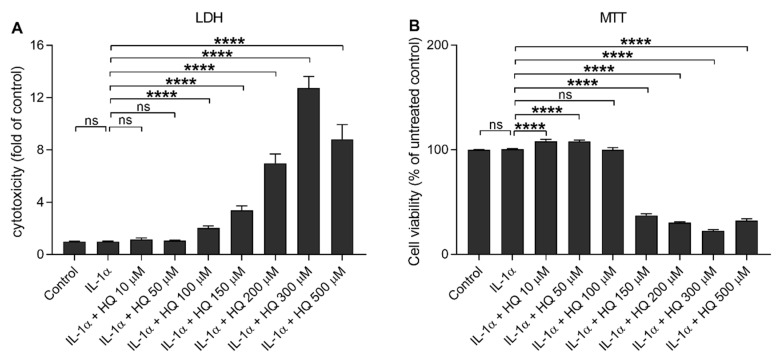
The effect of hydroquinone (HQ 10–500 μM; 18 h) on the cell viability of IL-1α-primed ARPE-19 cells measured using the LDH (**A**) and MTT (**B**) assays. Results have been normalized to the mean of the untreated control group. IL-1α-treated cells served as a control for hydroquinone exposure in primed cells. Data were combined from three independent experiments with four parallel samples per group. Results are shown as mean ± SEM. **** *p* < 0.0001, ns—not significant, Mann-Whitney U-test.

Hydroquinone induces ROS production and cytotoxicity concentration-dependently in human ARPE-19 cells [15,16,17]. In order to investigate whether hydroquinone could induce the secretion of proinflammatory cytokines, IL-1β and IL-18, we determined their extracellular levels as well as their correlation with cell viability after an exposure of IL-1α-primed ARPE-19 cells to different hydroquinone concentrations ranging from 10 μM to 500 μM. Low hydroquinone concentrations (10–50 μM) were well tolerated by RPE cells since LDH release and MTT values remained unchanged when compared to IL-1α-primed ARPE-19 cells without hydroquinone (Figure 1). LDH release was significantly increased in cells exposed to at least 100 μM hydroquinone, while metabolic activity was significantly reduced when the hydroquinone concentration was 150 μM or higher (Figure 1).

**Figure 2:**
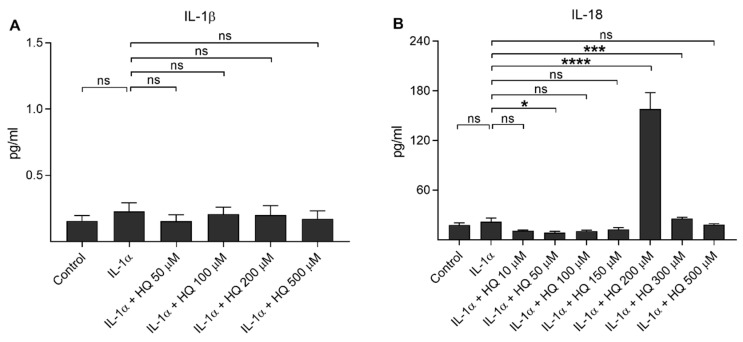
The effect of hydroquinone (HQ 10–500 μM; 18 h) on the release of IL-1β (**A**) and IL-18 (**B**) from IL-1α-primed ARPE-19 cells. Data were combined from three independent experiments with four parallel samples per group in each experiment. Results are shown as mean ± SEM. * *p* < 0.05, *** *p* < 0.001, **** *p* < 0.0001, ns—not significant, Mann-Whitney U-test. [Kruskal-Wallis test, (**A**) ns *p* = 0.903, (**B**) *** *p* < 0.001].

Next, we measured the production of proinflammatory cytokines IL-1β and IL-18 from IL-1α-primed ARPE-19 cells in response to hydroquinone exposure (Figure 2). Hydroquinone had no additional effect on the IL-1β release, which remained at the same level as in IL-1α-treated cells (Figure 2A). Hydroquinone induced significant secretion of IL-18 at the 200 µM and 300 µM concentrations, whereas lower (≤150 μM) or higher (500 μM) concentrations had no effect on the IL-18 levels (Figure 2B).

### 3.2. Hydroquinone Increases the Intracellular Level of NLRP3 in IL-1α-Primed ARPE-19 Cells

**Figure 3:**
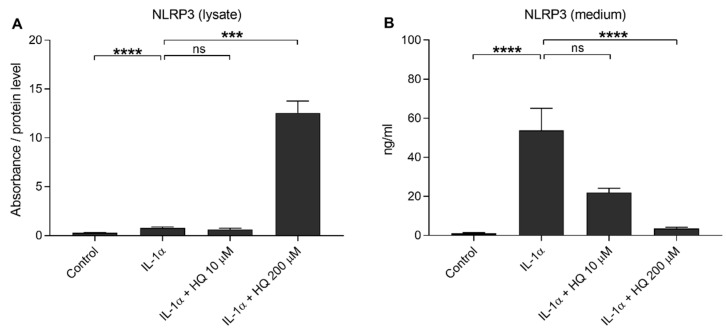
The effect of hydroquinone (HQ 10 μM, 200 μM; 18 h) on the levels of NLRP3 in cell lysate (**A**) and medium (**B**) samples in IL-1α-primed ARPE-19 cell cultures. Results (**A**) were normalized to the total protein level. Absorbance values in (**A**) were 0.263–0.507 in the control, 0.548–1.516 in the IL-1α, 0.416–0.862 in the IL-1α + HQ 10 μM, and 4.492–9.375 in the IL-1α + HQ 200 μM group. NLRP3 concentrations in medium samples (**B**) were 0.590–2.054 ng/mL in the control, 11.81–161.453 ng/mL in the IL-1α, 10.345–28.244 ng/mL in the IL-1α + HQ 10 μM, and 1.268–6.303 ng/mL in the IL-1α + HQ 200 μM group. Data were combined from three independent experiments with two (**A**) or three (**B**) parallel samples per group in each experiment. Results are shown as mean ± SEM. *** *p* < 0.001, **** *p* < 0.0001, ns—not significant, Mann-Whitney U-test.

We showed previously that the oxidative stress induced either by impaired protein clearance or 4-hydroxynonenal (HNE) exposure promoted NLRP3 inflammasome signaling with caspase-1 activation in RPE cells favoring the secretion of IL-1β or IL-18, respectively [14,18]. In the present study, we investigated whether hydroquinone promoted NLRP3 inflammasome activation in human ARPE-19 cells. We have also showed that NLRP3 is partially degraded, but also secreted, out of the cells after the activation of the inflammasome [37,38]. In the present study, IL-1α alone resulted in 2.8 and 43.9-fold intra- and extracellular NLRP3 levels, respectively, when compared to untreated control cells (Figure 3). Hydroquinone at the 10 μM concentration had no additional effect on the intra- or extracellular levels of NLRP3 when compared to IL-1α-treated cells without hydroquinone (Figure 3). In contrast, hydroquinone at the 200 μM concentration significantly elevated the levels of intracellular NLRP3, while the extracellular levels of NLRP3 remained significantly lower (Figure 3). In conclusion, we did not detect any evidence of the release of NLRP3 at the 200 µM hydroquinone concentration where the production of IL-18 peaked (Figure 2).

### 3.3. Hydroquinone Exposure Does Not Induce Caspase-1 Activation in ARPE-19 Cells

**Figure 4:**
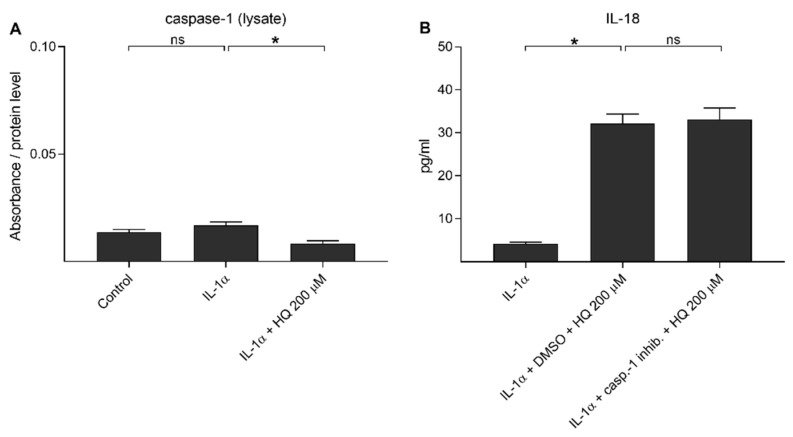
The effect of 200 μM hydroquinone (HQ; 18 h) on the cleaved caspase-1 (p20) levels detected from cell lysate (**A**) samples and the effect of caspase-1 inhibitor (casp.-1 inhib.; 20 μM in DMSO 0.22% *v/v*) on the hydroquinone-induced IL-18 release (**B**) in IL-1α-primed ARPE-19 cells. Absorbance values in (**A**) were 0.225–0.288 in the control, 0.259–0.379 in the IL-1α, and 0.149–0.161 in the IL-1α + HQ 200 μM group. Data were combined from two (**A**) or one (**B**) independent experiments with two (**A**) or four (**B**) parallel samples per group. Results are shown as mean ± SEM. * *p* < 0.05, ns—not significant, Mann-Whitney U-test.

Next, we investigated whether caspase-1 would be activated and contributes to the secretion of IL-18 upon hydroquinone exposure in IL-1α-primed RPE cells. Similarly to the situation with NLRP3, we previously observed that cleaved caspase-1 is partially released from the cell upon inflammasome activation [37,39]. IL-1α did not increase the intracellular levels of cleaved caspase-1 (p20) when compared to the untreated control cells (Figure 4A). Hydroquinone reduced the caspase-1 (p20) levels in cell lysate samples of IL-1α-primed ARPE-19 cells when compared to the primed cells without hydroquinone (Figure 4A). Cleaved caspase-1 was not detected in medium samples of controls or hydroquinone-treated cells. Absorbance values were 0.182–0.197 in the control, 0.193–0.204 in the IL-1α, and 0.182–0.193 in the 200 μM hydroquinone group of IL-1α-primed cells. Caspase-1 inhibitor had no effect on the IL-18 release (Figure 4B). These data suggest that hydroquinone did not activate caspase-1 in human ARPE-19 cells.

### 3.4. Hydroquinone Does Not Need Priming or NLRP3 Inflammasome Activation to Induce IL-18 Release

**Figure 5:**
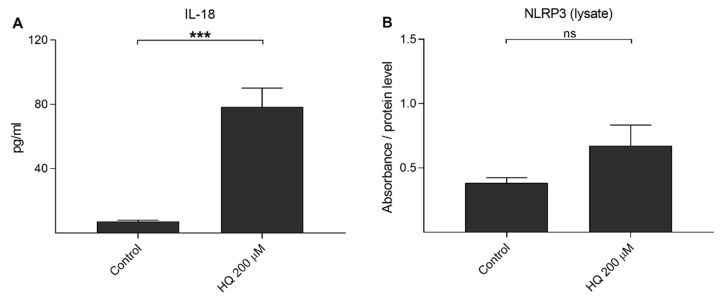
The effect of 200 μM hydroquinone (HQ; 18 h) on the IL-18 secretion (**A**) and on the intracellular NLRP3 level (**B**) in unprimed ARPE-19 cells. An untreated control group was used as the control for hydroquinone treatment. IL-18 concentrations in medium samples (**A**) were 4.552–10.556 pg/mL in the control and 32.132–123.617 pg/mL in the HQ 200 μM group. Absorbance values in (**B**) were 0.443–0.563 in the control and 0.345–0.579 in the HQ 200 μM group. Data were combined from two independent experiments containing four (**A**) or two (**B**) parallel samples per group. Results are shown as mean ± SEM. *** *p* < 0.001, ns—not significant, Mann-Whitney U-test.

**Figure 6:**
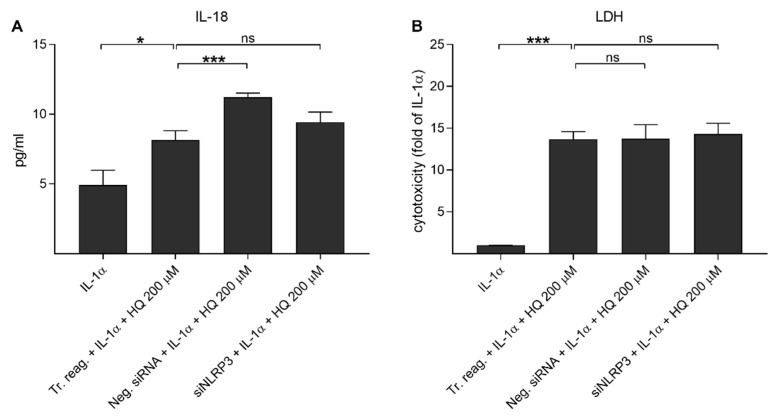
The effect of siNLRP3 on the hydroquinone-induced (HQ 200 μM; 18 h) release of IL-18 (**A**) and LDH (**B**) in IL-1α-primed ARPE-19 cells. Transfection reagent without siRNAs (Tr. reag.) was used as a control for the siNLRP3 treatment and negative siRNA (Neg. siRNA) was used to indicate if unspecific reduce of IL-18 release was detected. IL-18 concentrations in (**A**) were 1.342–8.686 pg/mL in the IL-1α, 4.267–11.166 pg/mL in the Tr. reag. + IL-1α + HQ 200 μM, 10.187–12.171 pg/mL in the neg. siRNA + IL-1α + HQ 200 μM, and 6.233–12.171 pg/mL in the siNLRP3 + IL-1α + HQ 200 μM group. Data were combined from two independent experiments with four parallel samples per group in each experiment. Results are shown as mean ± SEM. * *p* < 0.05, *** *p* < 0.001, ns—not significant, Mann-Whitney U-test. [Kruskal-Wallis test, (**A**) *** *p* < 0.001, (**B**) *** *p* < 0.001].

We have showed previously that UVB irradiation-induced IL-1β secretion was NLRP3-dependent, but this was not the case for IL-18 release [39]. Next, we examined whether hydroquinone alone would be capable of inducing the release of IL-18 without IL-1α-priming. Hydroquinone significantly increased IL-18 secretion but the levels of intracellular NLRP3 remained at the levels of untreated control cells (Figure 5). In order to confirm the NLRP3-independent IL-18 release, cells were treated with siNLRP3 before the hydroquinone exposure of IL-1α-primed cells. Specific NLRP3 siRNA did not reduce IL-18 release when compared to hydroquinone with transfection reagent without siNLRP3 in IL-1α-primed cells (Figure 6). These findings support the data shown above that hydroquinone was able to induce IL-18 secretion from ARPE-19 cells without the activation of NLRP3 inflammasome.

### 3.5. APDC Reduces the Release of IL-18 But Not NLRP3, While NAC Has the Opposite Effect upon Hydroquinone Exposure of IL-1α-Primed ARPE-19 Cells

**Figure 7:**
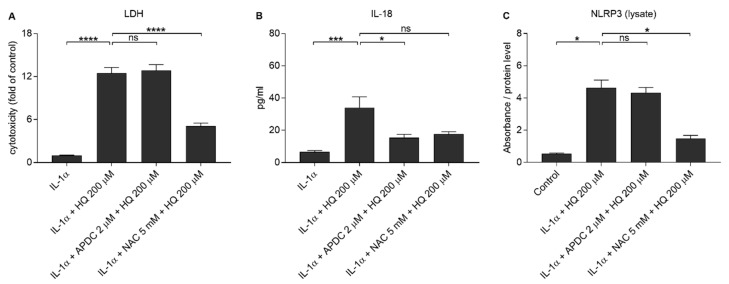
The effect of antioxidants (2 μM APDC, 5 mM NAC) in conjunction with 200 μM hydroquinone (HQ; 18 h) on the release of LDH (**A**) and IL-18 (**B**), and on the intracellular NLRP3 (C) levels in IL-1α-primed ARPE-19 cells. Data were combined from four (**A**,**B**) or 2 (**C**) independent experiments containing four (**A**, **B**) or two (**C**) parallel samples per group. IL-18 concentrations in (**B**) were 0.745–10.597 pg/mL in the IL-1α, 6.515–83.423 pg/mL in the IL-1α + HQ 200 μM, 2.58–26.253 pg/mL in the IL-1α + APDC + HQ 200 μM, and 7.721–26.506 pg/mL in the IL-1α + NAC + HQ 200 μM group. Absorbance values in (**C**) were 0.62–0.72 in the control, 2.171–2.824 in the IL-1α + HQ 200 μM, 1.816–2.564 in the IL-1α + APDC + HQ 200 μM, and 1.266–2.25 in the IL-1α + NAC + HQ 200 μM group. Results are presented as mean ± SEM. * *p* < 0.05, *** *p* < 0.001, **** *p* < 0.0001, ns = not significant, Mann-Whitney U-test.

**Figure 8:**
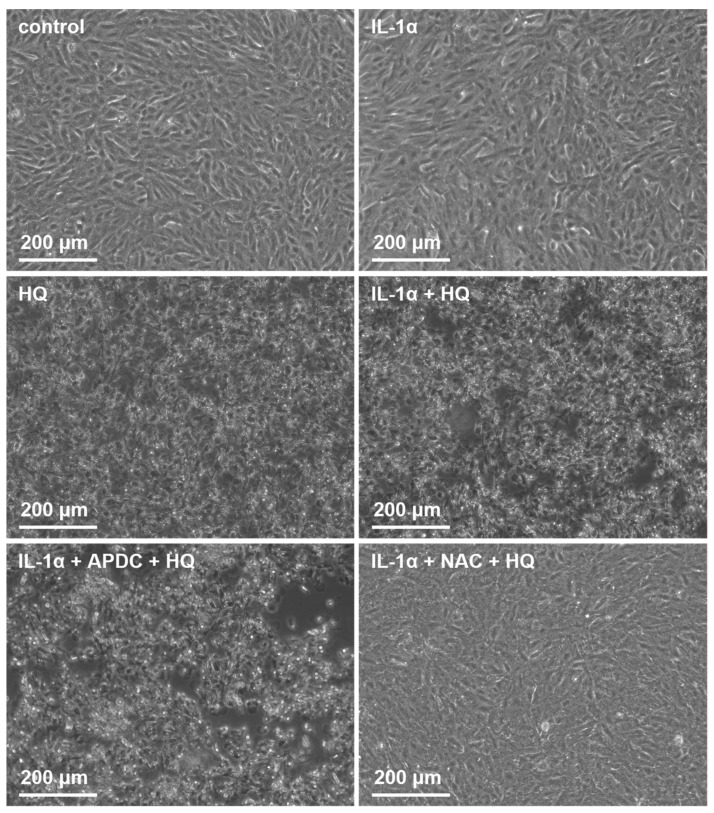
The effect of 200 μM hydroquinone (HQ; 18 h) on ARPE-19 cells with and without IL-1α-priming and antioxidants (2 μM APDC, 5 mM NAC). Cells were visualized using the Axio Vert A1 Zeiss microscope with AxioCam MRm camera and Zen 2011 program (Carl Zeiss Microscopy GmbH, Jena, Germany).

We showed previously that both NAC and APDC are able to reduce hydroquinone-induced ROS production in RPE cells [15]. Since it is known that IL-18 release from UVB-treated ARPE-19 cells is ROS-dependent [39], we tested whether antioxidants APDC or NAC would exert any effect on RPE cells exposed to hydroquinone. APDC had no effect on the LDH release, but NAC reduced it when compared to primed RPE cells after hydroquinone exposure without the supplementation with antioxidants (Figure 7). Microscope observations were in line with the LDH data since APDC-treated cells were damaged, but the NAC-treated cells appeared healthy and viable (Figure 8). APDC reduced the secretion of IL-18 but did not affect the intracellular NLRP3 levels (Figure 7). In contrast, NAC had no effect on the levels of IL-18 in IL-1α-primed ARPE-19 cells exposed to hydroquinone, but the levels of NLRP3 as a part of the total protein concentration were significantly lower when compared to the respective cells without NAC (Figure 7). These results suggest that the release of IL-18 and the expression of NLRP3 protein are not dependent on each other in the cells’ response to hydroquinone exposure.

### 3.6. Hydroquinone Increases the Levels of Extracellular ATP

**Figure 9:**
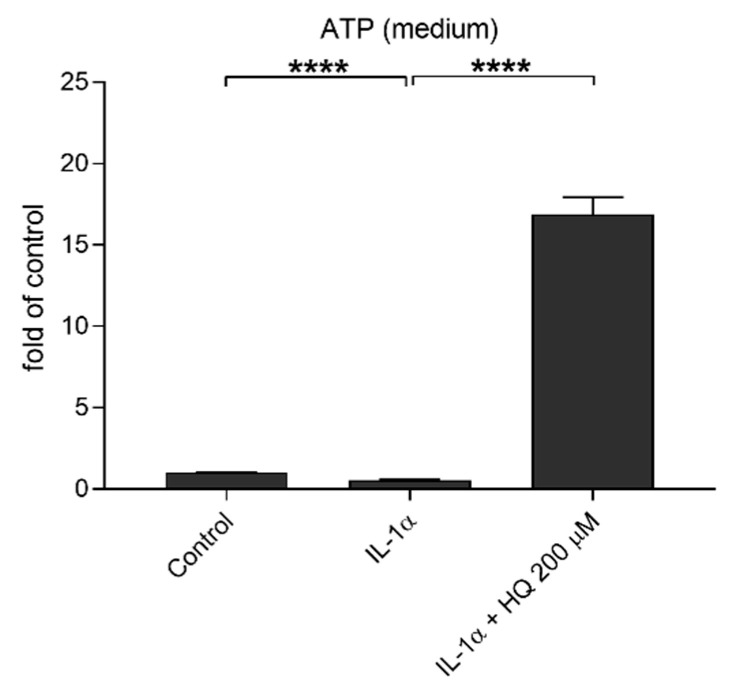
The effect of hydroquinone (HQ 200 μM; 18 h) exposure on the levels of extracellular ATP in IL-1α-primed ARPE-19 cell cultures. Results were normalized to the untreated control group. Data were combined from three independent experiments with four parallel samples per group in each experiment. Results are shown as mean ± SEM. **** *p* < 0.0001, Mann-Whitney U-test.

Extracellular ATP is reported to be one potential activator of the NLRP3 inflammasome with the effect mediated through the P2X7 receptor, but it can also serve as a secondary response to NLRP3 activation as well as a stimulant for the antioxidant system in times of oxidative stress [18,23,40]. In order to study the potential role of ATP, we measured the extracellular ATP levels after the IL-1α-primed cells were exposed to hydroquinone. Hydroquinone increased the extracellular ATP levels concurrently while elevating the intracellular level of NLRP3 and the amount of IL-18 from IL-1α-primed ARPE-19 cells (Figure 9). IL-1α treatment reduced the extracellular ATP levels in ARPE-19 cell cultures, whereas hydroquinone at 200 μM concentration significantly increased the extracellular ATP levels in IL-1α-primed cells (Figure 9).

### 3.7. Hydroquinone-Induced Reduction in PARP Levels Reflect DNA Damage in ARPE-19 Cells

**Figure 10:**
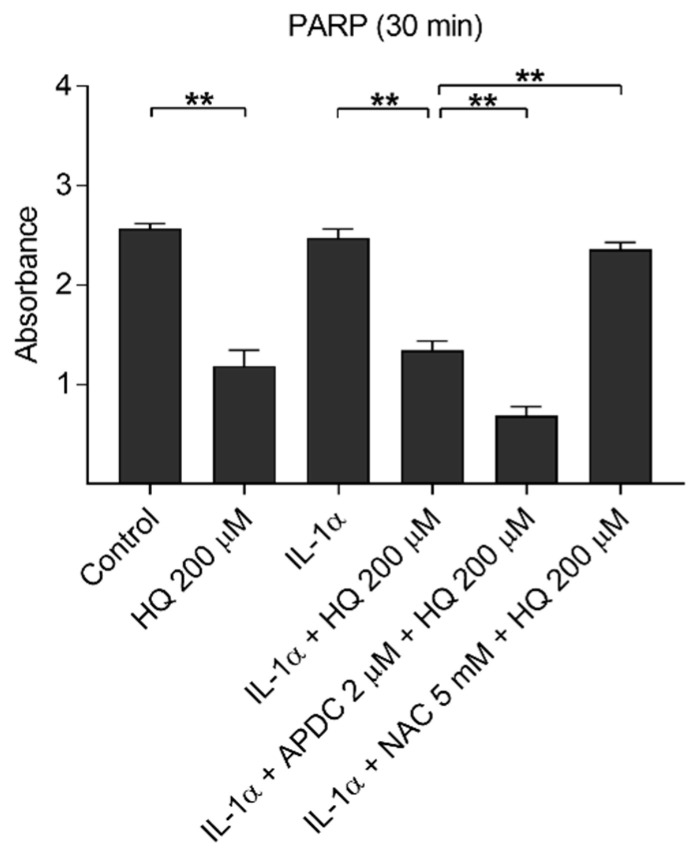
The effect of 200 μM hydroquinone (HQ) on the intracellular PARP level with and without IL-1α-priming and antioxidants (2 μM APDC, 5 mM NAC) in ARPE-19 cells. IL-1α and untreated control group were used as the control for hydroquinone treatment. Data were collected from two independent experiments containing three parallel samples per group. Results are shown as mean ± SEM. ** *p* < 0.01, Mann-Whitney U-test.

DNA damage induces NLRP3 inflammasome activation in human keratinocytes [41]. In order to investigate hydroquinone-induced DNA damage and to estimate the correlation between IL-18 release and DNA damage, we determined intracellular PARP levels after hydroquinone exposure. Hydroquinone significantly reduced the levels of the DNA-repairing PARP protein in ARPE-19 cells irrespective of the presence of IL-1α (Figure 10). APDC boosted the effect of hydroquinone with the reduction being statistically significant after 30 min exposure. In contrast, NAC treatment increased the PARP levels indicating that it was able to alleviate DNA damage.

## 4. Discussion

AMD is a consequence of RPE cell degeneration that causes photoreceptor cell death and impaired vision [7]. Oxidative stress, a major factor in the pathogenesis of both dry and wet AMD, is a potent activator of the NLRP3 inflammasome in RPE cells [7,12,13,14,18]. Hydroquinone is a genotoxic and cytotoxic compound that induces ROS production in RPE cells [15,16,17,42,43,44]. However, hydroquinone is sensitive to any kind of variation in experimental conditions, such as changes in diluent or medium supplements, which can influence the concentration needed to evoke cytotoxicity [16,17,43,45]. In the present study, signs of cellular membrane rupturing and reduced metabolic activity were evident in IL-1α-primed human RPE cells when the hydroquinone concentrations were greater than 100 μM and 150 μM, respectively. In our previous experiments with ARPE-19 cells without priming, the corresponding hydroquinone concentrations were 125 μM and 200 μM [15]. Thus, our data suggest that if ARPE-19 cells are exposed to the proinflammatory IL-1α cytokine, this makes them more sensitive to the adverse effects of hydroquinone. This finding is in line with the observation that treatment with an IL-1 receptor antagonist reduced hydroquinone-induced apoptosis in keratinocytes by preventing IL-1α activity [46].

NLRP3 inflammasome activation with subsequent caspase-1 activation is known to result in the cleavage of pro-IL-1β and pro-IL-18 into their mature and secreted forms [12,19,20,28]. However, NLRP3 inflammasome activation does not always lead to the secretion of both cytokines [33,39,47]. The accumulation of Alu RNA has been shown to induce IL-18 but not IL-1β release from RPE cells [33]. The outcome in ARPE-19 cells, especially of IL-18 production, appears to be highly context-dependent. HNE induced the secretion of both cytokines but impaired protein clearance favored IL-1β secretion with undetectable IL-18 levels [14,47]. Upon exposure to UVB, the same cell cultures produced both cytokines but only IL-1β was NLRP3 inflammasome-dependent [39]. The present study revealed one more difference i.e., hydroquinone induced IL-18 secretion but had no effect on the production of IL-1β in IL-1α-primed ARPE-19 cells. The propensity of hydroquinone to promote IL-18 production has also been observed in a mouse model of contact hypersensitivity [48], which supports the concept that the regulation of IL-18 release strongly depends on the activation signal and may not be as strictly inflammasome-dependent as is the case for IL-1β.

In our previous study, where UVB induced an NLRP3 inflammasome-independent release of IL-18, we observed that it was ROS that were responsible for the release of the cytokine [39]. We wished to examine another DNA-damaging factor and, therefore, we studied hydroquinone. We observed that APDC, but not NAC, reduced IL-18 secretion after the cells were exposed to hydroquinone. We previously showed that both the NADPH oxidase inhibitor APDC and the ROS scavenger NAC can reduce ROS production induced by hydroquinone in RPE cells [15]. The present data suggest that NADPH oxidase could contribute to the IL-18 production evoked by hydroquinone exposure. However, since NAC treatment exerted no response in RPE cells, APDC probably prevented IL-18 release by some other mechanism and not by inhibiting NADPH oxidase-mediated ROS production.

Extracellular ATP can protect cells from DNA damage caused by chemical exposure; it mediates this effect by activating P2X7 receptors, leading to NADPH oxidase-mediated activation of caspase-4 and 5 [42,44,49,50]. In human intestinal endothelial cells, caspase-4 has been shown to increase mature form of IL-18 in response to an exposure to enteric pathogens [51]. Our results are line with the concept that the ATP-induced NADPH oxidase mediated caspase-4 or 5 activation could contribute to the production of IL-18, since along with increased IL-18 production, hydroquinone resulted in significant ATP release from RPE cells. In addition, the ability of ATP release to alleviate DNA damage would be compatible with this concept since hydroquinone reduced the PARP levels in RPE cells. PARP participates in the DNA damage repair response and induces poly (ADP-ribose) (PAR) production-related DNA repair [52]. Hydroquinone has been shown to induce DNA damage in human hepatoma HepG2 and TK6 lymphoblastoid cells [42,53,54]. In the latter cells, the extent of the DNA damage correlated with immediately reduced PARP levels as well as with increased PAR production [42].

In this study, we investigated the potential of hydroquinone exposure to induce NLRP3 inflammasome activation in ARPE-19 cells. Hydroquinone induced DNA damage and promoted IL-18 secretion, which was independent from the NLRP3 inflammasome. Since IL-18 has been reported to reduce DNA damage in UVB-irradiated mouse keratinocytes [55,56], the present results do not exclude the probability that the release of both ATP and IL-18 in response to hydroquinone exposure would be attempts by the human RPE cells to alleviate hydroquinone-induced DNA damage. That proposal will need to be examined in further studies.

## 5. Conclusions

Hydroquinone induced DNA damage and promoted an NLRP3-independent IL-18 release from RPE cells. NADPH oxidase contributed to IL-18 release but the detailed mechanism still needs clarification.

## Data Availability

The data presented in this study are available on request from the corresponding authors.
